# Online inspection of blackheart in potatoes using visible-near infrared spectroscopy and interpretable spectrogram-based modified ResNet modeling

**DOI:** 10.3389/fpls.2024.1403713

**Published:** 2024-06-07

**Authors:** Yalin Guo, Lina Zhang, Yakai He, Chengxu Lv, Yijun Liu, Haiyun Song, Huangzhen Lv, Zhilong Du

**Affiliations:** ^1^ Chinese Academy of Agricultural Mechanization Sciences Group Co., Ltd., Beijing, China; ^2^ Key Laboratory of Agricultural Products Processing Equipment in the Ministry of Agriculture and Rural Affairs, Beijing, China; ^3^ China National Packaging and Food Machinery Corporation, Beijing, China

**Keywords:** visible-near infrared spectroscopy, modified ResNet, Grad-CAM++, online analysis, blackheart in potatoes

## Abstract

**Introduction:**

Blackheart is one of the most common physiological diseases in potatoes during storage. In the initial stage, black spots only occur in tissues near the potato core and cannot be detected from an outward appearance. If not identified and removed in time, the disease will seriously undermine the quality and sale of theentire batch of potatoes. There is an urgent need to develop a method for early detection of blackheart in potatoes.

**Methods:**

This paper used visible-near infrared (Vis/NIR) spectroscopy to conduct online discriminant analysis on potatoes with varying degrees of blackheart and healthy potatoes to achieve real-time detection. An efficient and lightweight detection model was developed for detecting different degrees of blackheart in potatoes by introducing the depthwise convolution, pointwise convolution, and efficient channel attention modules into the ResNet model. Two discriminative models, the support vector machine (SVM) and the ResNet model were compared with the modified ResNet model.

**Results and discussion:**

The prediction accuracy for blackheart and healthy potatoes test sets reached 0.971 using the original spectrum combined with a modified ResNet model. Moreover, the modified ResNet model significantly reduced the number of parameters to 1434052, achieving a substantial 62.71% reduction in model complexity. Meanwhile, its performance was evidenced by a 4.18% improvement in accuracy. The Grad-CAM++ visualizations provided a qualitative assessment of the model’s focus across different severity grades of blackheart condition, highlighting the importance of different wavelengths in the analysis. In these visualizations, the most significant features were predominantly found in the 650–750 nm range, with a notable peak near 700 nm. This peak was speculated to be associated with the vibrational activities of the C-H bond, specifically the fourth overtone of the C-H functional group, within the molecular structure of the potato components. This research demonstrated that the modified ResNet model combined with Vis/NIR could assist in the detection of different degrees of black in potatoes.

## 1 Introduction

Potatoes, vital vegetables in the human diet and for food security, are widely produced and consumed worldwide (Sanchez et al., 2020). Potatoes are always purchased as fresh tubers or processed food products such as potato flour, dehydrated potato flakes, frozen potatoes, French fries, and chips (Sampaio et al., 2021). During growth, harvesting, and post-harvest storage, a variety of factors such as insect bites, bacterial or fungal infections, cutting by harvesting knives, collision and extrusion, and changes in the post-harvest storage conditions can cause different potato defects, reducing the quality of the potatoes (Hajjar et al., 2021). Blackheart is one of the most common physiological potato diseases that can occur during storage and transport. In the beginning stages, discoloration occurs only in the tissues around the center of the potato, which is not visible from the outside. If not detected and promptly removed, this disease can severely affect the quality and sale of the entire potato batch. Therefore, detecting potato defects can not only help meet the different needs of end-consumers and maximize resource utilization but also allow potato producers and sellers to analyze the types of defects and adopt targeted strategies to improve production management (Kothawade et al., 2021). Therefore, finding a method of early blackheart disease detection in potatoes is crucial. Experts typically perform defect detection. However, these procedures are often time-consuming, labor-intensive, and limited by consistency and accuracy in judgment by different personnel. Hence, efficient and effective automated methods are needed to detect blackheart potato defects (Zhou et al., 2015).

Vis/NIR spectroscopy has been extensively used for the rapid detection and nondestructive control of quality characteristics of various agro-food products (He et al., 2022). Zhou Zhu et al. examined the potential of using Vis/NIR transmission spectroscopy in the 513–850 nm range, along with chemometric techniques such as partial least squares-linear discriminant analysis (PLS-LDA), to classify potatoes affected by blackheart in a static state. Height-corrected transmittance demonstrated the best performance, with the calibration and validation set achieving a 97.11% success rate (Zhou et al., 2015). The transmission spectra of 470 potatoes, including 234 healthy potatoes and 236 blackheart potatoes, were collected by Han et al. using the left-to-right transmission method. Based on the potato Vis/NIR transmittance spectroscopy grading line and PLS-DA method, a potato blackheart disease discrimination model was established, which had a significant effect on detecting blackheart disease. The area under the receiver operating characteristic curve (AUC) of the model, total discrimination accuracy, RMSECV, and RMSEP values were 0.994, 97.16%, 0.28, and 0.26, respectively, thus demonstrating that the transmission method could accurately and rapidly identify blackheart potatoes. The average spectral difference between blackheart and healthy potatoes reached a maximum at 705 nm (Ya-fen et al., 2021). Based on the principle of Vis/NIR diffuse transmission spectroscopy, Ding Jigang et al. carried out the simultaneous online nondestructive testing of blackheart disease and starch content by utilizing a non-destructive online inspection system using a self-designed laboratory system. The original spectra of 121 healthy potatoes and 116 blackheart potatoes in the 600–1000 nm band were averaged, and the results showed that the absorbance values of the blackheart potato samples in the 600–900 nm band were significantly higher than the healthy potato samples. The PLS-DA model blackheart potatoes achieved 97.89% accuracy with 97.74% and 98.33% correct calibration and validation sets. The model was implanted into an online detection system and externally validated using 50 samples not involved in modeling. The discrimination rate of potato blackheart disease was confirmed as 96% (Ji-gang et al., 2020). However, due to specially designed constraints and model parameters, the detection performance of models established by traditional algorithms, such as PLS-DA, may be limited (Rong et al., 2020).

Convolutional neural networks (CNNs) have been widely adopted in various fields, such as image recognition, natural language, and video processing. Vis/NIR spectroscopy combined with CNN models has been used to detect internal blackheart disease defects, achieving 98.2% accuracy (Wei et al., 2023). By blending NIR technology with 1D-CNN, a custom-built online spectral measurement system was used in this study to obtain the transmission spectra of 114 oranges in the range of 644–900 nm. The model was established by combining the diameter correction method (DCM) combined with 1D-CNN and demonstrated excellent performance. The recall values of the optimal model for unfrozen oranges and early freeze-damaged oranges were 88.54% and 95.15%, respectively, in the prediction set, with an overall accuracy of 91.96%. The proposed DCM and 1D-CNN methods could effectively eliminate the effect of size on the transmission spectra and allow the model to successfully identify freezing damage (Tian et al., 2022). As suggested by multiple studies, when the number of samples for analysis met specific requirements, a CNN combined with Vis/NIR could be applied for qualitative and quantitative analysis and obtain better analytical accuracy because the spectral response would have better wavelength accuracy and less external noise interference. However, research has been limited to improving discrimination accuracy, and the recognition mechanism of the CNN model has not been analyzed. To realize online real-time detection and understand the browning mechanism of Yali pears, Hao et al. conducted an online discriminant analysis on healthy Yali pears. Pears with different degrees of browning according to Vis/NIR spectroscopy showed that the prediction accuracy of the original spectrum combined with a 1D-CNN deep learning model reached 100% for the test sets of browned pears and healthy pears. A Gramian angular field (GAF) was also successfully used to transform the spectral data into graphs to further express and analyze the spectral features extracted by the 1D-CNN method (Hao et al., 2023). However, to the best of our knowledge, few studies have been performed on the performance of CNN model parsing with the qualitative analysis of blackheart potatoes while using CNNs.

To meet the requirements of online detection and understand the mechanism of blackheart in potatoes, the online detection feasibility of blackheart potatoes in top-to-bottom transmission mode was verified in this study. The specific objectives were as follows. (1) The complexity of the ResNet model was reduced by studying the modifications, in terms of the number of parameters, while aiming to improve predictive performance. (2) SVM, ResNet, and modified ResNet models were built and evaluated for their ability to discriminate between healthy and blackheart-affected potatoes. (3) Grad-CAM++ was employed to visually interpret the spectral features identified by the modified ResNet model. (4) The t-SNE technology was used to visualize the classification capabilities of different layers in a CNN.

## 2 Materials and methods

### 2.1 Potato samples

The potatoes(Xisen No.6) used in this experiment were purchased from a farmer’s supermarket in Beijing, and potato samples with surface damage and defects were removed. The equator diameter of the samples was measured by vernier calipers, where the height range was 48.1–59.8 mm, the average value was 53.9 mm, and the standard deviation was 3.62. To reduce the transmission spectrum affected by environmental factors, all potatoes were stored at ambient temperature for 24 h. Because no difference in appearance was observed between normal and blackheart potatoes, purchasing diseased samples directly in the market would require considerable effort. Therefore, potatoes with blackheart were artificially prepared in this experiment by inoculating the samples in an incubator and refrigerator. The main steps were as follows. The potatoes were cleaned and dried, packed in plastic bags after surface disinfection, and placed into an incubator at 38.5°C for 48 h, and then immediately placed into a refrigerator at 4°C for 48 h to prepare 1–4 grades of internally discolored potatoes (Ji-gang et al., 2020; Ya-fen et al., 2021).

### 2.2 Vis/NIR spectroscopy acquisition

**Figure 1:**
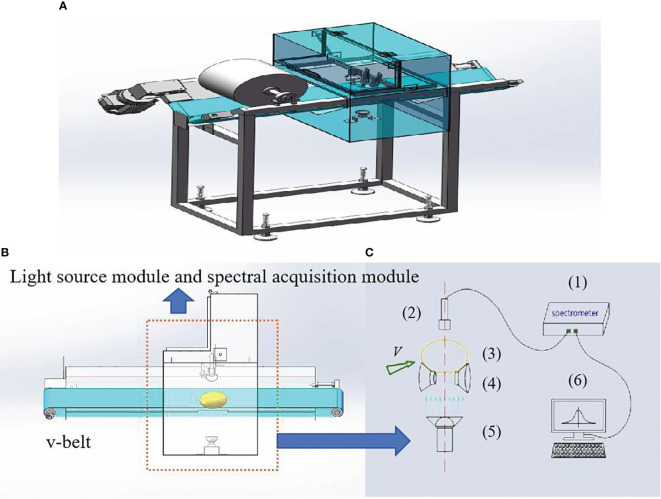
Vis/NIR transmission spectroscopy system: **(A)** three-dimensional figure; **(B)** cutaway view; **(C)** light source module and spectral acquisition module (1. Vis/NIR spectrometer; 2. probe; 3. sample; 4. tray; 5. light source; 6. computer).

Before spectral collection, the potatoes were equilibrated at room temperature for 4 h, and three spectra were collected from each potato. Spectral measurements of whole potato tubers were performed by a custom-build online transmittance spectral system, as shown in **Figure 1**. The system consisted of a Vis/NIR spectrometer (USB2000+, OceanOptics, USA), a 100 W tungsten halogen light source, and a convex lens that was installed at the front of the light source to focus the light on the surface of the potatoes. Potatoes were placed on the v-belt and moved forward at a speed of 0.5 m/s. Once the potatoes reached the light source, the light passed through the potato tissue and was collected by a detector located on the bottom, then transmitted by the fiber optics to the spectrometer. The spectrometer was then triggered to automatically save the spectra on the computer. The transmittance system captured light in the range of 350–1000 nm at an integration time of 100 ms. Each tuber was repeatedly scanned three times by the system, and all three measurements were used to determine the raw Vis/NIR spectra of the samples.

### 2.3 Evaluation of blackheart degree in potatoes

**Figure 2:**
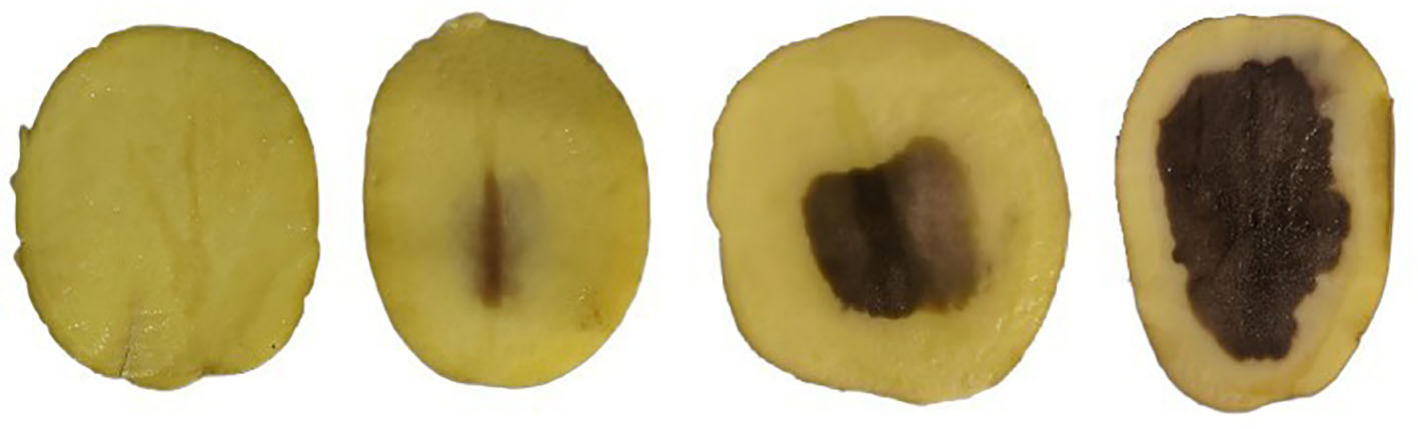
Example images of potatoes with different blackheart grades.

After spectra collection, the potatoes were cut along the long axis to record the degree of disease and whether discoloration occurred. Specifically, each potato was first cut in half along the longest axis, and then three experts with years of experience in potato detection determined whether the insides of the potatoes were black. The evaluated criteria are as follows (**Figure 2**). If the black center area was 0, the grade was 1; if the black center area was less than 10%, the grade was 1; if the black center area was 10–25%, the grade was 2; if the black center area was 25–50%, the grade was 3; if the black center area was greater than 50%, the grade was 4. After removing the undesirable data, 265 and 378 samples were divided into healthy and blackheart sets, with 150 samples for grade 2, 78 samples for grade 3, and 150 samples for grade 4.

### 2.4 Data augmentation of the spectra

Data augmentation (DA) techniques can artificially increase the dataset size and diversity to alleviate issues, thus enhancing model performance and generalization (Shorten and Khoshgoftaar, 2019). DA encompasses all employed methods to expand the number of samples in a dataset (Maharana et al., 2022). Using DA can increase the complexity of the training process, resulting in a more robust and accurate model compared with a model without DA (Wong et al., [[NoYear]]; Hernández-García and König, 2018). Moreover, DA techniques can help reduce costs and the complexities of optical spectroscopy data collection (Li et al., 2020), allowing them to find applications that include these tools for synthetic data generation (Gracia Moisés et al., 2023).

In this study, to fully train the SVM, ResNet, and modified ResNet models and improve network generalization performance and robustness, the experimental samples were reasonably expanded before model calibration by employing randomly adding Gaussian noise to enhance the diversity of sample data (Ma et al., 2021), increasing the total number of spectra from 643 to 3858.

### 2.5 Construction method of discriminant model

#### 2.5.1 SVM model

SVM serves as a discriminant classifier that can find the hyperplane with the greatest considerable minimum distance to the training data set, using quadratic programming optimization and a radial basis kernel (Fuentes et al., 2018). Regularization parameter gamma (γ), the radial basis function (RBF), kernel function parameter sig2 (σ^2^), and the penalty factor (*C*) are considered critical factors that can determine stability and performance. In this study, *C* was 1.0, and gamma served as the scale (Cen et al., 2016).

#### 2.5.2 ResNet model

**Figure 3:**
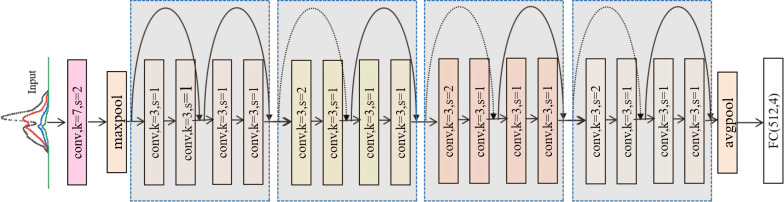
Example of a residual network with 18 parameter layers, with dotted shortcuts increasing the dimensions.

**Figure 4:**
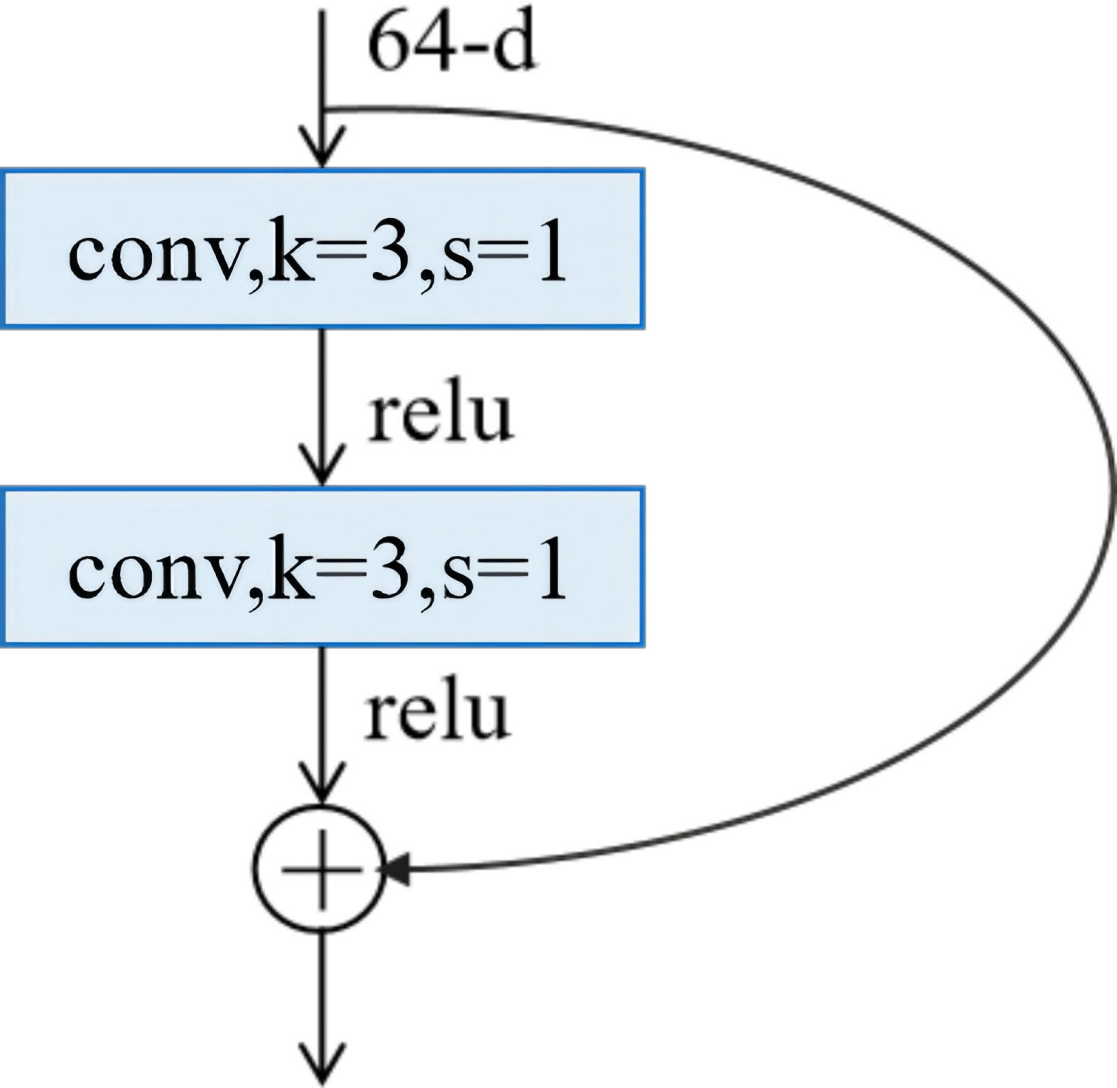
A residual unit.

Deep residual networks (ResNets) (**Figure 3**) were first introduced by He et al. (2016a) and are considered one of the most significant deeplearning architectural innovations in recent years. ResNets utilize residual unit (RU) blocks (**Figure 4**) stacked into modularized architectures.

An RU can be expressed by Equations 1, 2


(1) $$y_{l}=\text{ R}\left(x_{l},\;W_{l}\right)+h\left(x_{l}\right),\;$$

(2) $$x_{l}+l\;=\text{A}\left(y_{l}\right),\;$$

where *y_l_* and *x_l+1_* serve as the output and input of the *l*-th unit, respectively, and *W_l_* is a set of weights and biases of the *l*-th RU, which contains *K* layers. During training, the network aimed to learn each RU’s residual function R(*x_l_*, *W_l_*), with function h(*x_l_*) serving as the identity mapping type chosen for skip connection, and A was a non-linear activation function, as described in reference (He et al., 2016b).

#### 2.5.3 Depth-wise convolution and pointwise convolution

**Figure 5:**
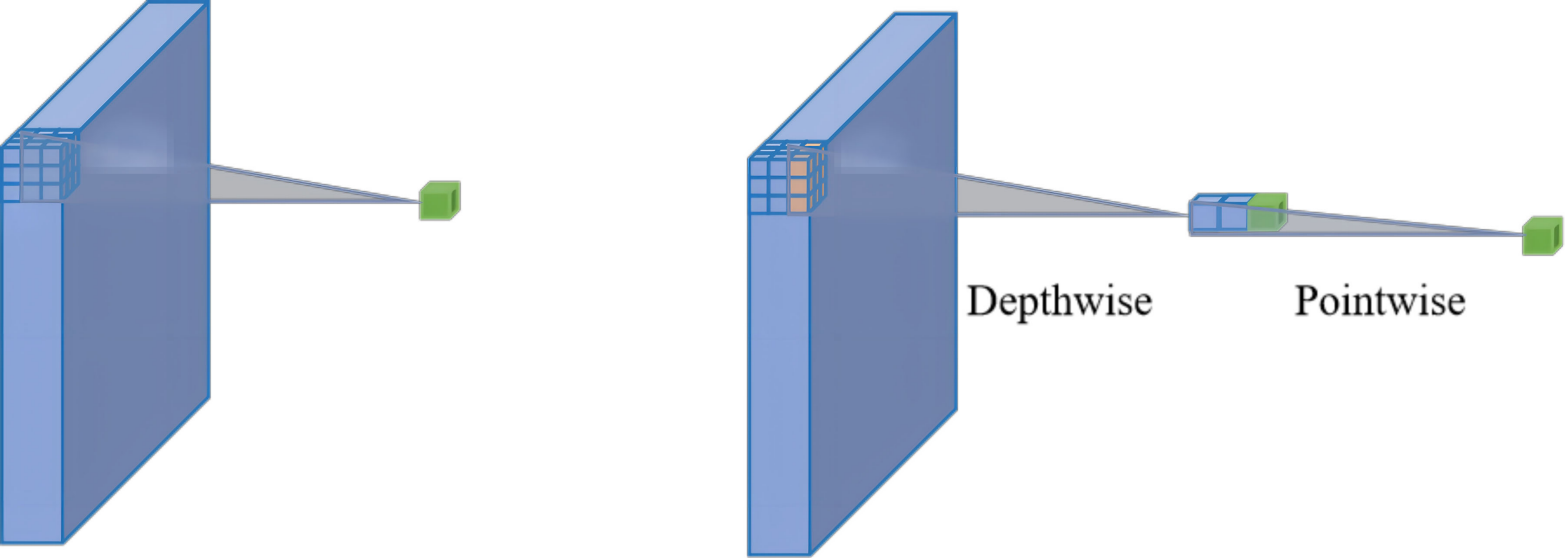
Standard convolution and depth-wise separable convolution.

Depth-wise separable convolution, based on depth-wise separable convolution, can divide a standard 3 × 3 convolution into 3 × 3 depth-wise convolution and 1 × 1 pointwise convolution. Although standard convolution can perform channel-wise and spatial-wise computation in one step, depth-wise separable convolution can split the computation into two steps, namely, depth-wise convolution can be applied to a single convolutional filter per each input channel, and depth-wise convolution output can be linearly combined using pointwise convolution. A comparison of standard convolution and depth-separable convolutions is shown in **Figure 5**. Depth-wise convolution and pointwise conjugation play different roles in generating new features, with the former used to capture spatial correlations, and the latter used to capture channel-wise correlations (Guo et al., 2019).

#### 2.5.4 Efficient channel attention for deep CNNs

**Figure 6:**
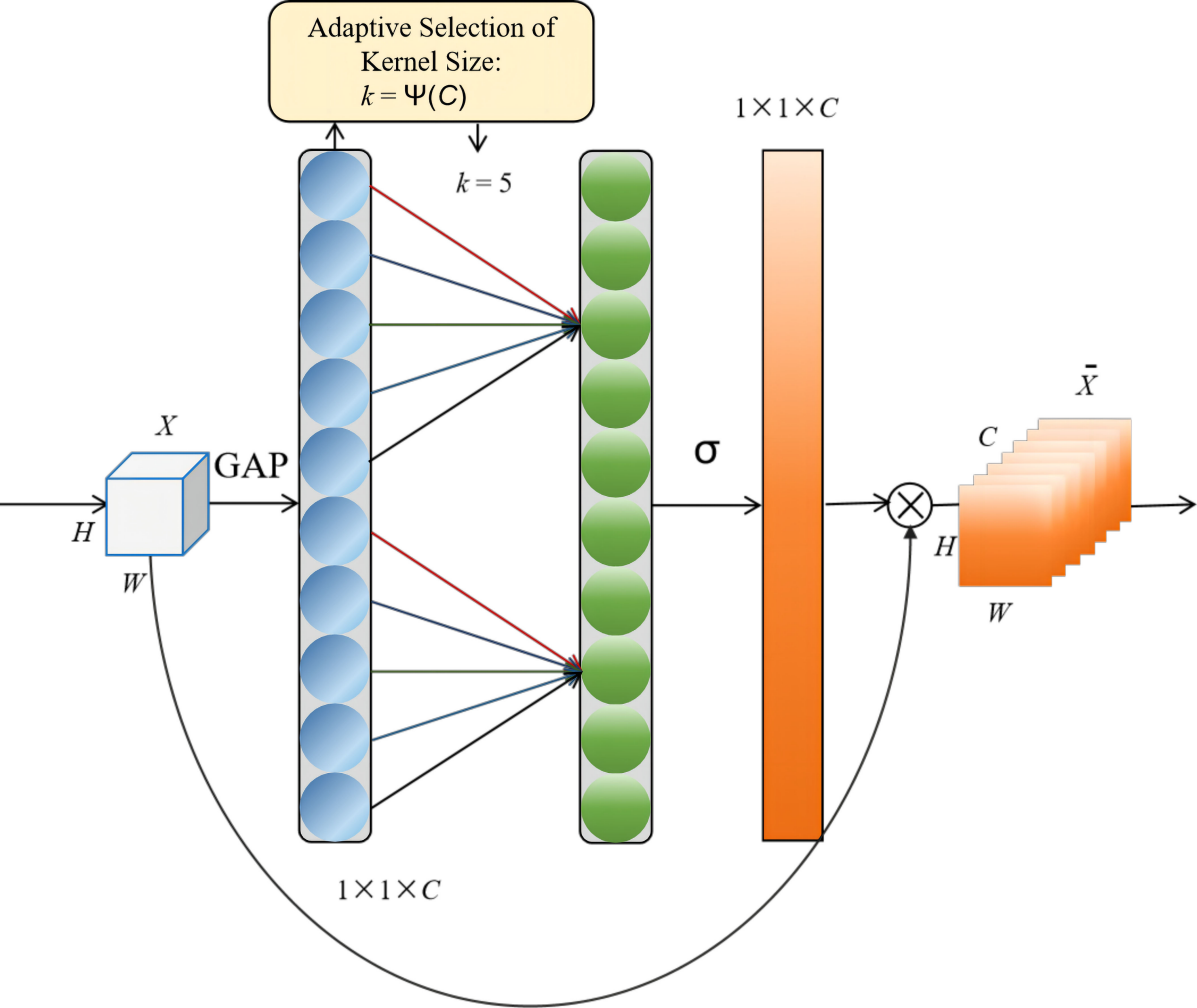
Diagram of efficient channel attention (ECA) module. Given the aggregated features obtained by global average pooling (GAP), ECA generated channel weights by performing a fast 1D convolution of size k, where k was adaptively determined via the mapping of channel dimension C.

For deep CNNs, the efficient channel attention (ECA) module, which avoided dimensionality reduction and efficiently captured cross-channel interaction, was proposed. As shown in **Figure 6**, ECA captured local cross-channel interaction after channel-wise global average pooling and without dimensionality reduction by considering each channel and each channel’s *k* neighbors. This methodology has been shown to guarantee both efficiency and effectiveness. ECA could be efficiently implemented by size k in fast 1D convolution, where the kernel size, denoted by *k*, represented the extent of local cross-channel interactivity coverage, i.e., how many neighbors were included in the attention prediction of a channel. To avoid the manual tuning of *k* via cross-validation, a method to adaptively determine *k* was developed, where the interaction coverage (i.e., kernel size, k) was proportional to the dimension of the channel (Wang et al., 2020).

#### 2.5.5 Modified ResNet model

In this work, we proposed a modified ResNet model based on one-dimensional Vis/NIR spectral data to more accurately determine whether the potatoes were subjected to blackheart disease.

**Figure 7:**
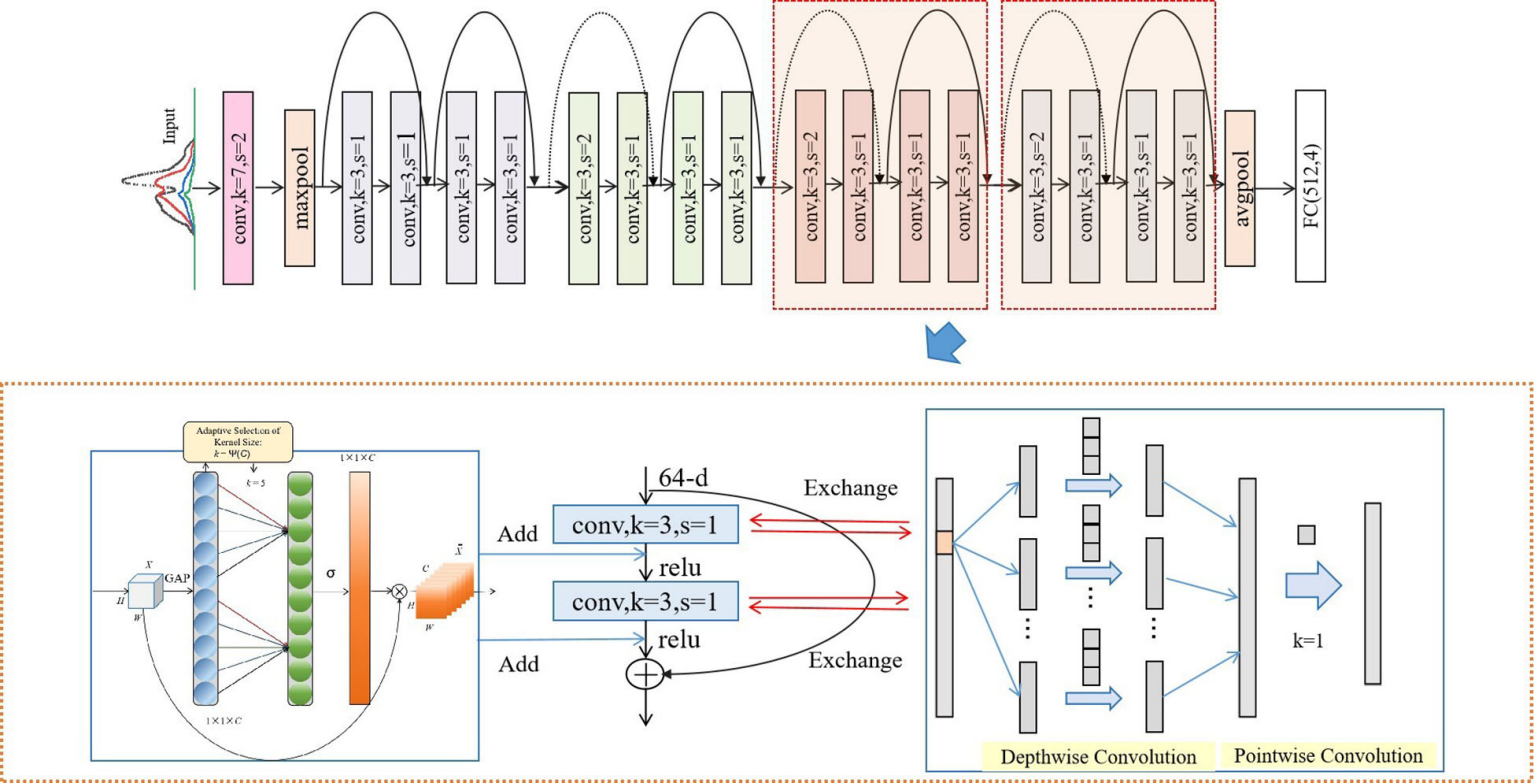
Diagram of the modified ResNet model.

The structure of the modified ResNet model (**Figure 7**) was similar to that of the ResNet-18 model. Unlike the ResNet-18 model, several standard convolutions in the ResNet-18 were replaced with depth-wise separable convolutions, specifically in layers with more than 128 channels, to significantly reduce the computational complexity and the number of parameters. In the modified ResNet-18, ECA layers were applied after batch normalization in each basic block, but only in layers with more than 128 channels, focusing the model’s attention where it was most beneficial while keeping the computational load manageable. The decision to use depth-wise separable convolutions and ECA layers only in layers with more than 128 channels demonstrated a strategic approach to balance computational efficiency with model performance. This adaptive adjustment ensured that these enhancements were applied in deeper layers where the complexity and number of channels increased and where the optimizations had the most significant impact. An Adam optimizer was selected for the ResNet and modified ResNet model, which automatically adjusted the learning rate during the training process, thereby enhancing convergence speed and reducing the need for manual adjustment. The initial learning rate for the Adam optimizer was set at 0.01, and a weight decay (L2 regularization) coefficient of 1×10^−4^ was introduced to mitigate the possibility of model overfitting. The model’s training lasted for 100 epochs and the batch size was 256, during which the model underwent one forward pass and one backward pass through the entire training dataset in each epoch to update the model parameters.

### 2.6 Explanation of models

The Grad-CAM++ visualization method has been widely applied, with a basic premise that the feature map corresponding to a particular classification can be expressed as a gradient, and the global average of the gradient can be utilized to calculate the weight (Zhang et al., 2022). In addition, ReLU and the weight gradient were added to the feature map. Only one back propagation was required to calculate the gradient, which was originally applied to 2D but improved and applied to 1D signals by Zhang et al (He et al., 2023). 

### 2.7 Evaluation of the models

The dataset was divided into three sets for different purposes, where 80% of the data was allocated to train the model, 10% was used to validate the model, and the remaining 10% was used to test the model’s performance. The overall accurate identification rate (accuracy) was adopted to evaluate the online discriminative model of blackheart potatoes, with accuracy referring to the correct identification rates and classifiers for all samples. Specifically, the greater the values of these indexes, the higher the accurate classification rates.

The experiment was implemented in PyTorch 2.1.0 and Python 3.9, and Origin 2024 (Origin Lab Corporation, Northampton, MA, USA) was used to construct the graphs. A Windows 10 64-bit operating system carried out all software operations, as the software platform, with an Intel(R) Core i7–6700HQ CPU 3.40GHz (8 GB of RAM).

## 3 Results

### 3.1 Vis/NIR spectral analysis of potatoes

**Figure 8:**
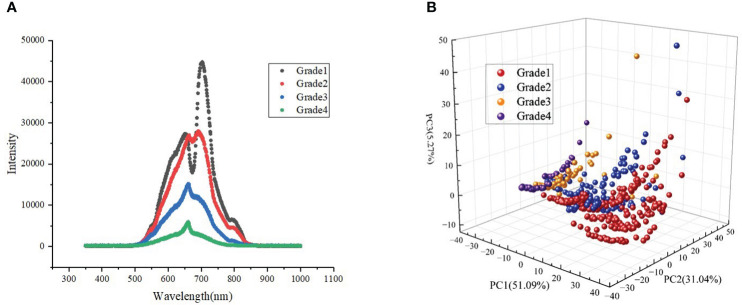
Vis/NIR spectra of potatoes with different degrees of blackheart **(A)** and spatial distributions of the first three principal components of potato samples with different degrees of blackheart **(B)**.

During transmission, the discoloration of the potato flesh increased light absorption within the tissue, and the loss of water in the tissue could lead to increased light scattering within the tissue (Sun et al., 2016), resulting in higher light absorption and lower transmittance. As shown in **Figure 8**, the transmission intensity values of the mean spectra of different grades of blackheart potatoes in the range of 500–850 nm were significantly lower than healthy potatoes. However, the mean spectral curves of grade 1 and grade 2 blackheart potatoes were approximately coincident in the range of 500–650 nm, and these potatoes were located in upper grades 3 and 4. Between 650 and 850 nm, the spectral transmission intensity decreased as the degree of black center increased. The average spectral differences between the black-centered potatoes and healthy potatoes reached local maxima near 650, 703, and 798 nm, with a maximum near 703 nm, indicating that the difference between the spectral values of black-centered potatoes and healthy potatoes was the greatest near 703 nm. In addition, the peak at around 650 nm was possibly the wavelength associated with chlorophyll, where the peak at about 700 nm potentially resulted from the stretching and contraction of the fourth overtone of the C–H functional group. Meanwhile, the peak at around 800 nm was possibly related to the stretching and contraction of the third overtone of the N–H functional group (Zou et al., 2010).

PCA can effectively reduce the spectral dimension while retaining representative information. In this study, the spatial distribution of potato spectra with different degrees of blackheart was analyzed by applying PCA, and the cumulative contributions of the first three principal components were 51.09%, 82.13%, and 87.40%, respectively (**Figure 8**). Although there was some overlap between the spectra of the samples collected from different blackheart degrees, the spatial distribution of the main components demonstrated little similarity, indicating significant differences between the sample spectra collected by four different blackheart degrees.

### 3.2 Four-class classification by full wavelengths

**Figure 9:**
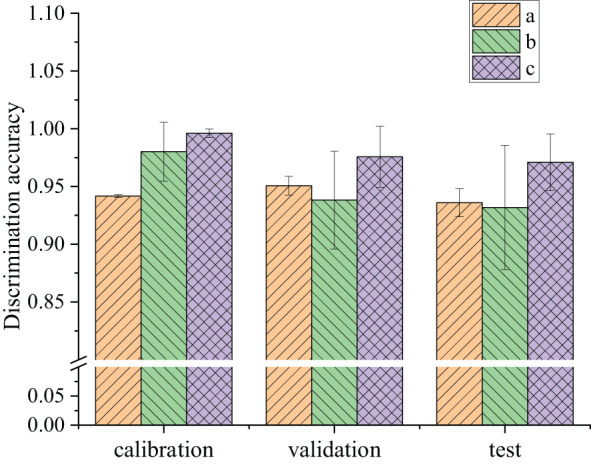
Discrimination accuracy of the calibration sets, validation sets, and test sets in 10 SVM, ResNet, and modified ResNet models: (a) SVM; (b) ResNet; (c) modified ResNet.

The SVM, ResNet, and modified ResNet discriminant methods were separately used to build online models to identify healthy and blackheart potatoes. These models were then used to qualitatively discriminate between healthy and blackheart potatoes, which were not included in the models. Each experiment was repeated 10 times to avoid the influence of chance. The discrimination results of the calibration sets, validation sets, and test sets in the 10 SVM, ResNet, and modified ResNet discriminant methods for potatoes are shown in **Figure 9**. The modified ResNet had better discrimination performance than ResNet and SVM, as demonstrated by the improved validation and test accuracy in almost all runs.

Specifically, the modified ResNet Model emerged as the most effective, increasing accuracy in the range of 0.989–1 in the calibration sets shown in **Figure 9**, indicating a robust balance between high efficiency and consistent outcomes. Its performance peaked at perfection (1.0) in at least one instance, underscoring its potential for optimal results. By contrast, the SVM model, despite its lowest accuracy performance (range of 0.939–0.943), showcased the highest consistency across all runs. The standard ResNet model, while outperforming SVM in accuracy, suffered from the highest variability in the results. This inconsistency pointed to its sensitivity to training set variations, which could entail a risk of significant underperformance in specific scenarios, as highlighted by its lowest performance mark (0.910–0.996). In conclusion, the modified ResNet model stood out as the superior choice for tasks, requiring both high accuracy and consistency in calibration sets.

In the validation sets, the SVM model emerged with a commendable average accuracy in the range of 0.940–0.966, characterized by its low variability (standard deviation of 0.00815), indicating a strong and consistent performance across different validation sets. However, the ResNet model showed a more comprehensive range of performance, with a range of 0.847–0.995, but a significantly higher standard deviation of 0.0424, indicating the potential for high performance but with the risk of significant inconsistency. This variability highlighted the importance of careful tuning and validation to ensure optimal performance across different datasets. The modified ResNet model exhibited the highest accuracy range of 0.907–0.995, though with a notable standard deviation of 0.0263. This suggested that while it generally outperformed the other models in terms of effectiveness, its results showed some degree of variability.

Analysis of the performance metrics for the SVM, ResNet, and modified ResNet models over 10 runs on the test sets provided insightful observations regarding their ability to generalize to new, unseen data. As shown in **Figure 9**, the SVM model showed an accuracy range of 0.909–0.951, suggesting that while the model was generally reliable, there was a slight variation in its effectiveness across test sets, with a standard deviation of 0.0121, indicating relatively consistent results across runs. The performance of the ResNet model showed an average performance, highlighted by a performance range of 0.811–0.992, but with a higher standard deviation of 0.0537, which was the largest of the three models. This significant variability suggested that the ResNet model could achieve exceptional highs, but also notable lows, indicating its sensitivity to the specifics of the test data. The modified ResNet model had an excellent performance range of 0.917–0.992, demonstrating its superior ability to handle test sets with better consistency than ResNet, albeit with some variability(0.0245).

**Table 1** shows the average results of 10 parallel runs of the SVM, ResNet, and modified ResNet models for potato quality assessment across calibration, validation, and test sets. These data allowed for a detailed comparison of the effectiveness of each model and its ability to generalize. The SVM model improved slightly from the calibration set (0.942) to the validation set (0.951) before experiencing a slight drop in the test set (0.936). This indicated that the SVM model not only was robust but also slightly improved or maintained its predictive ability across different stages, demonstrating good generalization to unseen data. The ResNet model showed higher performance for the calibration set (0.980), but then declined slightly in performance for the validation (0.938) and test (0.932) sets. This pattern suggested that while ResNet performed exceptionally well on the calibration set, its ability to generalize to unseen data declined slightly. The modified ResNet model was identified as outperforming the other models for all three datasets. Its performance exhibited only minor declines from the calibration (0.996) to the validation (0.976) and test sets (0.971), not just maintaining high-performance consistency, but also demonstrating exceptional learning and generalization capabilities. This model’s slight performance decline across different datasets was minimal, underscoring its robustness and effectiveness in handling both seen and unseen data, thus making it the superior model among the three.

**Table 1:** The average results of 10 parallel runs of the SVM, ResNet, and modified ResNet discriminant models for potato quality.

Models	Calibration sets	Validation sets	Test sets
SVM	0.942	0.951	0.936
ResNet	0.980	0.938	0.932
Modified ResNet	0.996	0.976	0.971

The ResNet and modified ResNet models were compared, as shown in **Table 2**, focusing on the number of parameters, parameter reduction, and accuracy improvement. The ResNet model, with 3,845,956 parameters, served as the baseline for this comparison. The parameters of the modified ResNet model were significantly reduced to 1,434,052, achieving a substantial 62.71% reduction in model complexity. The reduction of parameters increased the efficiency of the modified ResNet model in terms of computational resources, thus improving its performance, as demonstrated by the 4.18% increase in accuracy. This analysis demonstrated the effectiveness of the modifications made to the ResNet model. By simplifying the architecture, the modified ResNet model became more efficient in terms of resources and also improved its predictive performance. Optimizing deep learning models could significantly improve their efficiency and effectiveness, making them crucial for applications requiring high accuracy without significant parameter computational burden.

**Table 2:** Comparative analysis of the ResNet and modified ResNet models.

Model	Number of parameters	Parameter reduction	Accuracy improvement
ResNet	3845956	/	/
Modified ResNet	1434052	62.71%	4.18%

### 3.3 Visual analysis

**Figure 10:**
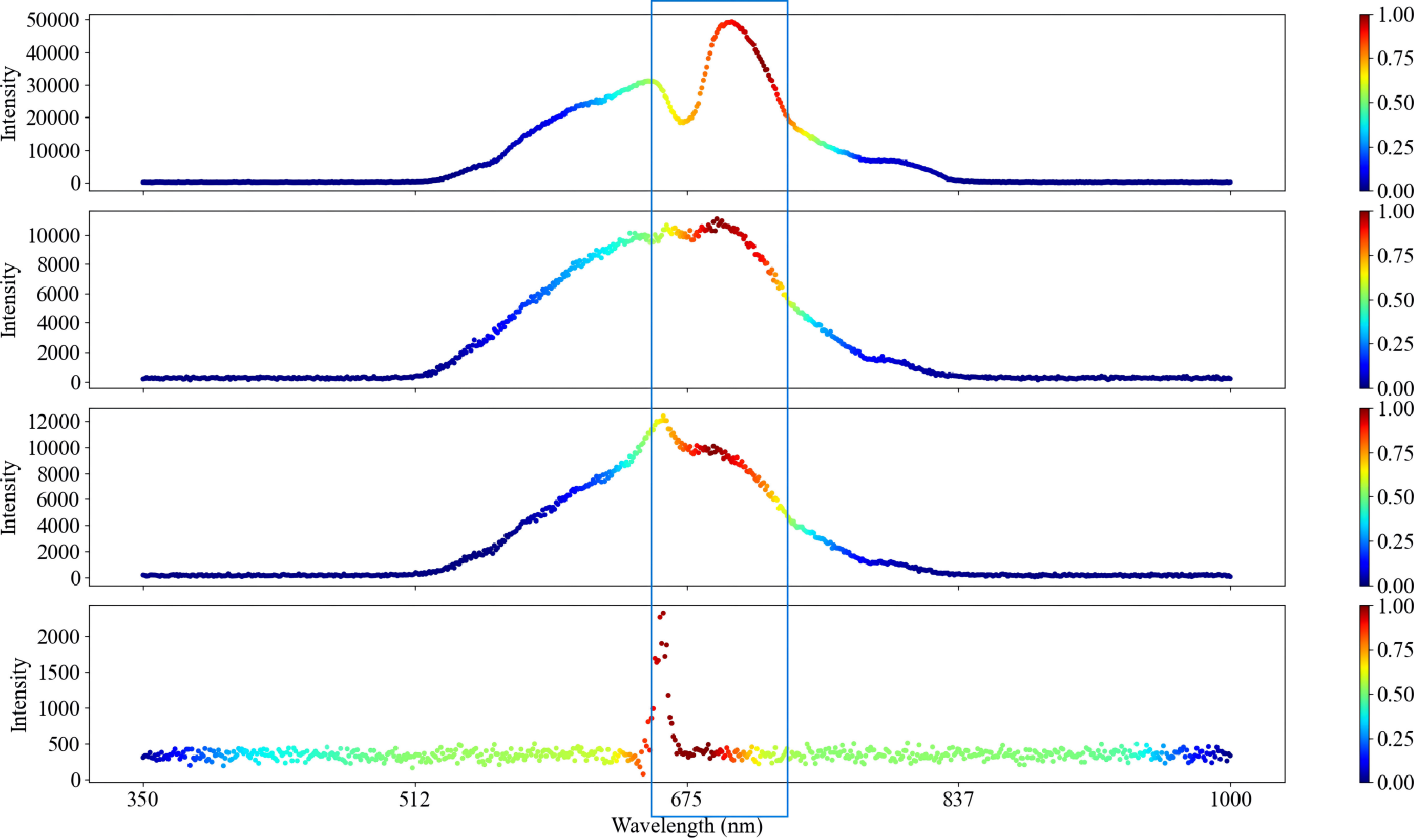
Visualization of four blackheart states under Grad-CAM++ (from top to bottom: grade 1, grade 2, grade 3, grade 4).

To further express and analyze the spectral features extracted by the modified ResNet method, Grad-CAM++ was used to visualize the spectral data weight values. The Grad-CAM++ encoding process of the potato spectral data is shown in **Figure 10**. The blue-to-red color represented the importance of the wavelength, where the closer the color to red, the higher the degree of activation, and the higher the feature importance. Conversely, the closer the color to blue, the lower the degree of activation, and the lower the feature importance. As shown in the figure, the red region was mainly concentrated between 650 and 750 nm, which reached a local maximum near 700 nm.

**Figure 11:**
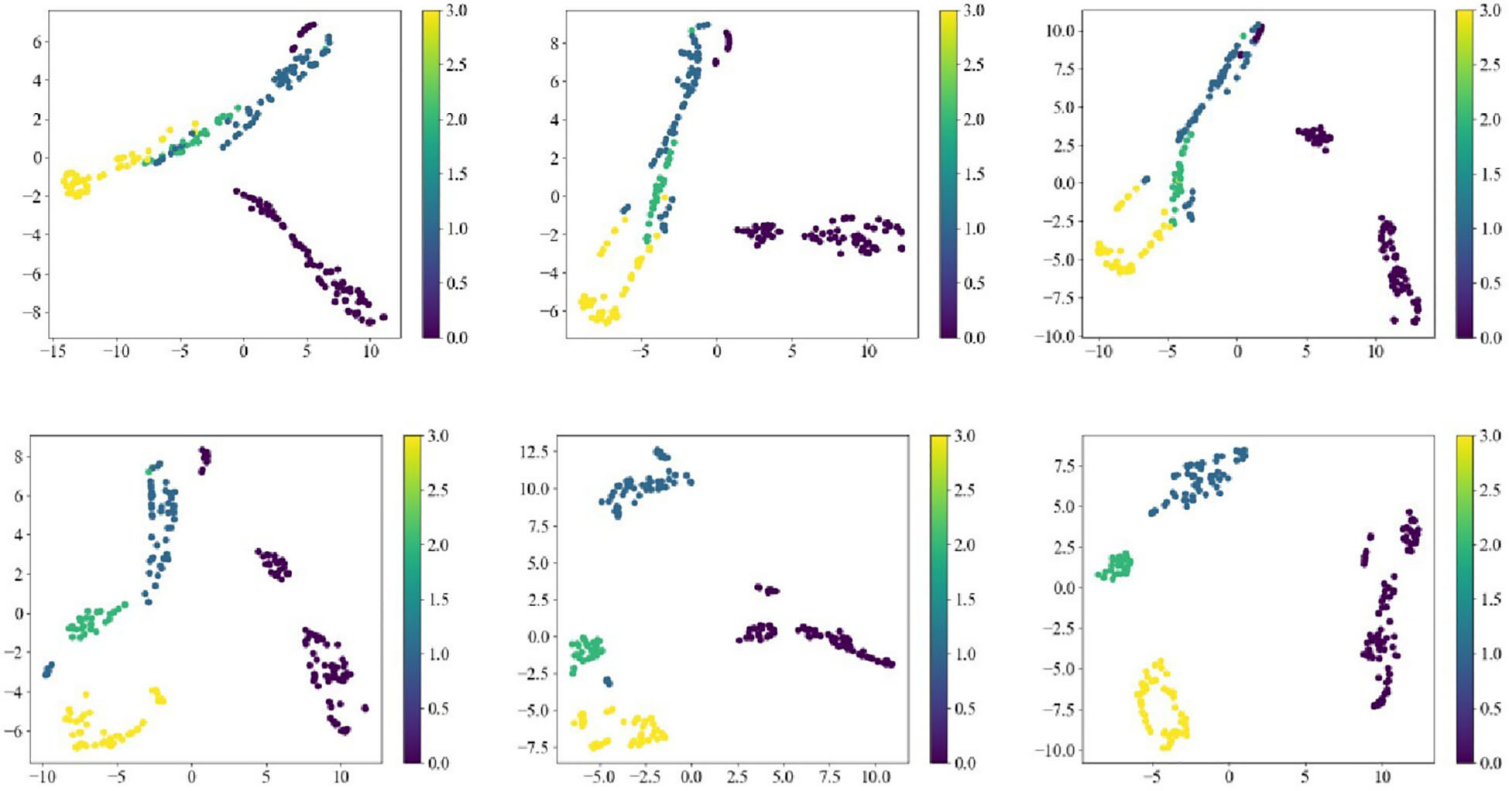
Feature visualization of different layers (from top to bottom and from left to right: conv1, layer1, layer2, layer3, layer4, average pooling).

The t-distributed stochastic neighbor embedding (t-SNE) technique has been especially useful for visualizing high-dimensional datasets, as it can translate the high-dimensional data into a lower-dimensional space and visualize the clustering and separation of the data points. In this study, the t-SNE technology was applied to feature visualization and further reveal the feature representations, with different colors representing different grades. **Figure 11** shows a collection of t-SNE visualizations representing the spatial distributions of different layers in a convolutional neural network, ranging from conv1 to subsequent layers (layer1, layer2, layer3, layer4, average pooling). In conv1, the features exhibited minimal separation, where the spectral points of the three types of samples overlapped each with other, as this layer typically captured fundamental patterns and textures. From layer 1, and advancing to deeper layers, an evident increase in separation was observed, signifying that the network started to establish more defined groupings of features, possibly representing more intricate patterns. Further stratification was observed with the emergence of distinct clusters, and layer 2 likely discerned more complex features instrumental in differentiating various classes or data types. The clusters became more dispersed, potentially reflecting a refinement in feature discrimination in layer 3. Layer 4 was well-defined, though more scattered and contained clusters, suggesting an advanced level of abstraction and feature discernment. In this stage, the network likely pinpointed the most critical features for the task that it was trained to accomplish. The t-SNE plot for the average pooling layer often showed a clear feature distinction between the different categories, possibly because this layer helped to reduce the spatial dimensions and summarize the essential features detected by previous layers. Each visualization captured the intricate structure of the data and reflected the network’s ability to learn discriminative features at various levels of abstraction, thus confirming that deep learning models have a powerful capability to comprehend and process complex datasets (van der Maaten and Hinton, 2008).

In summary, these visualizations provided insight into how a CNN processed and transformed input data into increasingly clear categorization. From conv1 to average pooling, increasing separation and distinct clustering with progression to deeper layers indicated the network’s ability to distinguish between increasingly abstract features. This demonstrated the network’s ability to extract and refine features necessary for performing complex pattern recognition tasks.

## 4 Discussion

The Vis/NIR spectral analysis revealed critical spectral features that are key in distinguishing healthy potatoes from those affected by blackheart disease. The model’s high attention to wavelengths between 650–750 nm, particularly around 700 nm, underscores the importance of these spectral regions in identifying the biochemical changes associated with the disease (Ya-fen et al., 2021). These findings align with prior research indicating the significance of the C-H bond’s fourth overtone in disease identification (Zou et al., 2010). The Grad-CAM++ visualizations further validated these findings by highlighting these specific wavelength regions as critical for accurate disease detection. The features identified by the modified ResNet model combined with Grad-CAM++ were similar to those found by Zhou et al. (2015)(678, 698, 711, 817, 741, and 839 nm) and Han et al (Ya-fen et al., 2021)(658, 665, 668, 675, 688, 695, 705, 712, 732, 740, 800, 810, 810, 816, and 839 nm), where 66.67% and 66.67% of the researched blackheart feature bands included by the modified ResNet model combined Grad-CAM++ selected feature areas, respectively.

In the study, the modified ResNet model consistently outperformed the SVM and traditional ResNet models in terms of both accuracy and reliability across different datasets. This superior performance can be attributed to the architectural enhancements in the modified ResNet, including depth-wise and pointwise convolutions and efficient channel attention modules. These modifications not only reduced the model’s computational load by significantly cutting down the number of parameters (62.71% reduction) but also improved its ability to capture and process spectral data more effectively. The improvements in model architecture led to a notable increase in accuracy (up to 4.18%), which is crucial for applications that require high precision such as the online detection of blackheart in potatoes. In addition, a discrimination accuracy of 0.971, slightly higher than previous related studies of 96.68% (Zhou et al., 2015) and 96.73% (Ya-fen et al., 2021), was achieved on the test set without requiring feature extraction.

The early detection of blackheart disease facilitated by the modified ResNet model could significantly mitigate agricultural economic losses by reducing crop waste and improving storage and quality control measures. This technological advancement aligns with sustainable agriculture practices by promoting the efficient use of resources and minimizing the impact of diseases on food security. Despite the promising results, the study faces challenges such as generalizing the findings to other potato varieties or similar diseases in different crops.

## 5 Conclusion

This research has demonstrated that the modified ResNet model, integrated with Vis/NIR spectroscopy, is highly effective in the early diagnosis and real-time detection of potato blackheart disease. By incorporating depth-wise and pointwise convolutions coupled with efficient channel attention modules, the modified ResNet model demonstrated exceptional accuracy and achieved this while significantly reducing the complexity of its parameters. The model effectively distinguishes between healthy and blackheart-affected potatoes by focusing on critical spectral features, particularly in the 650–750 nm range, with a notable peak at 700 nm. This model’s non-invasive, accurate, timely detection capabilities highlight its potential to transform potato quality assessment and disease management, contributing to sustainable agricultural practices. Future work should expand the model’s application to other potato varieties and diseases, broadening its utility in the agricultural sector.

## Data availability statement

The original contributions presented in the study are included in the article/supplementary material. Further inquiries can be directed to the corresponding author/s.

## Author contributions

YG: Writing – original draft, Writing – review & editing. LZ: Writing – original draft, Writing – review & editing. YH: Investigation, Supervision, Writing – review & editing. CL: Investigation, Supervision, Writing – review & editing. YL: Software, Validation, Writing – review & editing. HS: Software, Validation, Writing – review & editing. HL: Funding acquisition, Resources, Writing – review & editing. ZD: Resources, Supervision, Writing – review & editing.
